# A successive framework for brain tumor interpretation using Yolo variants

**DOI:** 10.1038/s41598-025-13155-4

**Published:** 2025-07-31

**Authors:** S. Priyadharshini, Ramasubramanian Bhoopalan, D. Manikandan, Krishnaraj Ramaswamy

**Affiliations:** 1 Department of Electronics and Communication Engineering, SRM TRP Engineering College, Tiruchirappalli, Tamil Nadu 621105 India; 2 Department of Mechanical Engineering, SRM TRP Engineering College, Tiruchirappalli, Tamil Nadu 621105 India; 3 Department of Mechanical Engineering, College of Engineering and Technology, Dambi Dollo University, Dambi Dollo, Ethiopia; 4 Center for Global Health Research, Saveetha Institute of Medical and Technical Sciences, Saveetha University, Chennai, India

**Keywords:** Brain tumor segmentation, Detection, Deep learning, Magnetic resonance imaging, YOLOv11, Engineering, Electrical and electronic engineering

## Abstract

Accurate identification and segmentation of brain tumors in Magnetic Resonance Imaging (MRI) images are critical for timely diagnosis and treatment. MRI is frequently used to diagnose these disorders; however medical professionals find it challenging to manually evaluate MRI pictures because of time restrictions and unpredictability. Computerized methods such as R-CNN, attention models and earlier YOLO variants face limitations due to high computational demands and suboptimal segmentation performance. To overcome these limitations, this study proposes a successive framework that evaluates YOLOv9, YOLOv10, and YOLOv11 for tumor detection and segmentation using the Figshare Brain Tumor dataset (2100 images) and BraTS2020 dataset (3170 MRI slices). Preprocessing involves log transformation for intensity normalization, histogram equalization for contrast enhancement, and edge-based ROI extraction. The models were trained on 80% of the combined dataset and evaluated on the remaining 20%. YOLOv11 demonstrated superior performance, achieving 96.22% classification accuracy on BraTS2020 and 96.41% on Figshare, with an F1-score of 0.990, recall of 0.984, mAP@0.5 of 0.993, and mAP@ [0.5:0.95] of 0.801 during testing. With a fast inference time of 5.3 ms and a balanced precision–recall profile, YOLOv11 proves to be a robust, real-time solution for brain tumor detection in clinical applications.

## Introduction

Even in wealthy nations, brain cancer is a common and deadly illness that takes many lives each year. Every year, brain cancer claims the lives of around 20,000 people in the US^1^. The hallmark of this illness is unchecked cell proliferation and dissemination. The body often uses cell division to create new cells to replace damaged or ageing ones. Tumours can develop as a result of damaged cells proliferating out of control when this process goes wrong. While secondary brain tumours spread from other body areas^2^, primary brain tumours start and stay inside the brain^3,4^. There are two types of tumours: benign and malignant^5^. While benign tumours do not spread or infiltrate adjacent tissues, malignant tumours do so and go on to develop new tumours elsewhere. Benign tumours can get large enough to produce severe symptoms and even be lethal, although both types might not return following excision.

The brain, which is crucial for making decisions and is at the centre of the neurological system, has to be protected from harm, and tumours are a serious risk. Meningiomas, gliomas, and pituitary tumours are among the brain tumours caused by abnormal cell growth. Usually benign, meningiomas develop in the membranes that surround the brain^6,7^. Although they are not malignant, they make up 36.1% of all primary brain tumours and can have serious consequences including convulsions and blindness. Because they come from glial cells instead of neurones, gliomas can range in severity and are frequently cancerous. Pituitary tumours, which are located near the base of the skull, can impact several biological systems including hormone control.

Recent developments in imaging have improved radiologists’ ability to identify brain tumours. As a non-invasive technique, MRI is essential for evaluating tumours. However, MRI scan interpretation requires certain knowledge and expertise, which may not always be available, particularly in environments with limited resources. As a result, diagnoses are either overlooked or delayed^8^.

Medical image analysis has been transformed by DL and computer vision. In tasks including diabetes detection, classification, and segmentation, CNNs in particular have demonstrated remarkable performance^9–12^, and^13^. However, real-time deployment is a challenge for many CNN-based CAD solutions. While segmentation approaches need substantial computer resources because of the intricacy of mask construction, lighter models frequently fail to effectively localise tumours^14^.

To overcome these constraints, a number of object detection techniques have been investigated. Prominent models that strike a compromise between computational cost and performance include Single Shot Multibox Detector (SSD)^15^, R-CNN^16,17^, and Fast R-CNN^18^. More recently, it has been suggested that self-supervised DL models combine positional and semantic data to increase accuracy^19,20^.

Because of its unified design and real-time inference capabilities, the YOLO family of models has attracted a lot of interest^21^. YOLO predicts bounding boxes and class probabilities in a single run by treating object identification as a regression job. This is especially helpful in medical settings where it’s critical to identify abnormalities like brain tumours quickly and accurately.

This study suggests a unique and effective framework that uses YOLOv9, YOLOv10, and YOLOv11 to perform both brain tumour identification and segmentation in order to solve the aforementioned issues.

The key contributions of this work are:

1. A comprehensive comparative evaluation of three recent YOLO variants applied to medical imaging;
2. Integration of intensity normalization, contrast enhancement, and edge-based ROI extraction to improve localization accuracy;
3. A segmentation-aligned detection pipeline that enhances performance on MRI data while maintaining real-time feasibility;
4. Extensive validation using two benchmark datasets — Figshare and BraTS — with analysis of detection accuracy, segmentation quality, and inference time.

The remaining paper is organized as follows: “Literature review” reviews related work and highlights existing gaps. “Methodology” presents the proposed methodology. “Results and discussion” reports experimental results. “Comparative analysis of segmentation models” discusses future research directions, and “Conclusion” concludes the study.

## Literature review

Diagnosing brain tumours in real-time is challenging, but deep learning (DL) algorithms show promise using digital images like EEG, CT, and MRI scans. DL models applied to MRI and CT scans have demonstrated improved accuracy. Researchers favor CNNs for brain tumour detection and classification, with models based on CNNs^6,22,23^, RNNs^24^, AEs^25^^26–28^. and hybrid methods^29,30^ offering superior accuracy for early diagnosis. Using the BrainMRNet dataset of 253 images (155 tumour, 98 normal), a model achieved 96% accuracy, 92% precision, and 96% sensitivity^31^. Similarly, a 3D CNN with an.

FFNN, trained on BraTS 2015, 2016, and 2018 datasets, achieved accuracies of 98%, 96%, and 92%, respectively, though performance varied by dataset.

In^32^, a patch-based DNN for brain tumour classification was validated across eight datasets, including MICCAI, ISLES 2015, 2017, and BraTS 2012–2015, achieving a 99.8% Similarity Coefficient. In^33^, an automated DNN and segmentation strategy combining local and global information achieved 80% sensitivity, 93% specificity, and an 85% dice score. In^34^, a hybrid CNN-based DL model achieved a dice score and sensitivity of 0.86, and specificity of 0.91. In^35^, a DSS-based model for brain tumour multi-classification using metaheuristic algorithms for feature selection achieved 95% accuracy on BraTS 2018 and 2019 datasets.

Several studies have explored the BT dataset for brain tumour classification^36–39^. In^36^, a CNN enhanced with factorised bilinear and dual suppression encoding achieved 95% accuracy^37^. proposed a TL-based residual network, with ConvNet, AlexNet, and VGG16 models confirming 95% accuracy. In^40^, Faster R-CNN achieved 77.60% precision, while^41^ reached 96.56% with a CNN, and^42^ integrated a gray-level co-occurrence matrix trained CNN, achieving 96.5%. However, the lack of clear task specifications and missing factors like recall methods and detection thresholds hinder deep analysis. Table 1 compiles key studies, tools, technologies, and models for tumour classification and detection.

**Table 1:** An overview of the current state of the art in brain tumor classification.

Refs. no.	Dataset details	Experimental techniques	Algorithms	Findings	Research gap	Summary	Future research
^23^	Private MRI dataset with labelled brain tumors	Data augmentation, attention-enhanced detection	Improved YOLOv8 with attention modules	Achieved high accuracy and detection speed	Limited validation on public datasets; lacks modality diversity	Introduces improved YOLOv8 for accurate brain tumor detection	Apply to multimodal and multi-institutional datasets
^24^	Publicly available MRI scans (not specified)	Instance segmentation; region-level analysis	YOLOv8 for segmentation	Effective instance segmentation with good IoU	Lack of tumor type classification; limited benchmarks	Explored YOLOv8 for medical instance segmentation	Combine segmentation with classification in hybrid models
^25^	BraTS 2020 dataset	Dilated convolutions for deep feature extraction	YOLOv8 with dilated conv backbone	Improved detection precision; better small tumor capture	High computational overhead; needs faster inference	Combines spatial features via dilation in YOLOv8	Optimize for real-time deployment; lightweight models
^26^	100 + studies across various medical domains	Meta-analysis, categorization by use-cases	YOLOv1–YOLOv8 evolution overview	YOLO models widely adopted in healthcare	No practical experimentation; review-centric	YOLO for medical object detection - A Systematic review	Conduct benchmarking studies across datasets and tasks
^43^	BraTS 2018 + synthetic datasets	Label-free synthesis with weak supervision	LF-SynthSeg model	High segmentation accuracy without labels	Limited testing on real-world unseen cases	Label-free segmentation through data synthesis	Improve generalization on heterogeneous clinical data
^44^	3-class MR image dataset (HGG, LGG, no tumor)	Transfer learning, fine-tuning CNNs	Fine-tuned EfficientNet	High accuracy and AUC for tumor detection	Poor interpretability; no segmentation capability	Demonstrates strength of EfficientNet on medical tasks	Combine EfficientNet with segmentation networks
^45^	Review covering > 50 papers (2015–2023)	Taxonomy of DL approaches	DL models (CNNs, GANs, RNNs, YOLO, etc.)	DL models outperform classical methods	Lack of standard benchmarks across studies	Broad survey of DL models for brain tumor tasks	Push toward unified evaluation protocols
^46^	Clinical MRI dataset	Morphological filtering + CNN	Robust automatic detection model	Accurate, noise-resistant segmentation	Not validated on large-scale public datasets	Morphological pre-processing improves detection	Validate on diverse and multicentric data
^47^	BraTS 2021 + monomodal T1/T2 images	Attention fusion; multimodal architecture	Multimodal segmentation with monomodal guides	Enhanced segmentation using healthy brain priors	Complexity and training time; inference cost	Monomodal input boosts multimodal segmentation performance	Compress architecture for clinical deployment
^48^	Br35H dataset (public), with data augmentation	Transfer learning, adjustable learning rate, custom callbacks	EfficientNet-B4	Achieved 99.87% accuracy; excellent sensitivity, specificity, and F1-score on augmented dataset	Focuses only on classification; no segmentation or real-time performance	Efficient and accurate tumor classification using pre-trained EfficientNet-B4	Extend to include segmentation and evaluate real-time performance in clinical settings
^49^	Br35H dataset	Comparative study using multiple optimizers (SGD, Adam, RMSprop)	AlexNet, LeNet, VGG16/19, ResNet50	AlexNet + SGD achieved 98.79% accuracy, 98.82% F1-score; effective sensitivity/specificity	Only classification; lacks segmentation, detection, and speed optimization	Benchmarks CNN architectures for tumor classification	Combine top CNN classifiers with real-time object detection and segmentation methods

Several studies have explored YOLO-based models for brain tumour diagnosis^50–53^, mostly using older versions like YOLOv5 and YOLOv7. These studies focus on detection but lack segmentation and direct comparisons across advanced variants. Hossain et al.^54^ used YOLOv5 with a microwave imaging system for brain abnormality detection, but it couldn’t perform segmentation, limiting tumour border identification. Shelatkar and Bansal^55^ applied YOLOv5 with transfer learning on BraTS 2020, but without comprehensive evaluation of detection and segmentation. Our research is the first to evaluate YOLOv9, YOLOv10, and YOLOv11 on the Figshare Brain Tumour (BT) dataset^56^.

Our study benchmarks three advanced YOLO models: YOLOv9 with anchor-free detection and Transformer-based feature extraction, YOLOv10 with a hybrid CNN-Transformer backbone, and YOLOv11 with multi-scale attention and deformable Transformer blocks. Unlike prior work, which typically adapts a single YOLO version with limited focus on comparative robustness^23,24^, our comparison highlights architectural advancements and clarifies the strengths and weaknesses of each for brain tumour analysis.

Yao et al.^23^ improved YOLOv8 for brain tumour detection with specialized modules but did not test multiple YOLO versions or achieve clinical-grade segmentation. Our study fills this gap by aligning segmentation masks with detection, using the BT standard format for boundary annotation, and evaluating various parameters. Unlike previous YOLO-based research, we emphasize real-time clinical deployment, combining speed, accuracy, and segmentation quality assessments^57,58^, setting a new benchmark with top algorithm evaluations and enhanced performance metrics.

Using DNN and the segmentation approach, automated methodology^59^ for identifying brain tumours were proposed. The BraTS 2013 dataset was utilised to train the suggested model using MR images. The model investigated a novel approach to diagnosis that uses both local and global information. Their model’s dice score was 85%, its specificity was 93%, and its sensitivity was 80%. To improve the identification of different items in photos, a number of object detection algorithms have been created^15,60^. The Single Shot Multibox Detector (SSD)^61^, R-CNN^62^, and Fast R-CNN^63^ are notable algorithms. The use of self-supervised deep learning models to improve performance by utilising aggregated semantic and positional data is also explored in some recent publications^19,64^. The YOLO algorithm^65^, on the other hand, has drawn a lot of interest because of its remarkable object identification system and use of a single, unified neural network. By approaching object detection as a regression issue and explicitly calculating bounding box coordinates and class probabilities from pixel-level data, this technique transformed object detection. With improved speed and accuracy, it allows for the simultaneous prediction of multiple bounding boxes and class probabilities.

Using EfficientNet-B4 with customisable learning rate scheduling and custom callbacks, recent research^48^ has shown the significant potential of deep CNNs and transfer learning in brain tumour classification tasks, attaining an impressive 99.87% accuracy on an upgraded Br35H dataset. Using 5-fold cross-validation, their method also demonstrated excellent sensitivity (99.97%), specificity (99.74%), and F1-score (99.66%). However, segmentation and real-time performance are not addressed by the model; it only concentrates on classification. Khushi et al.^49^ used the Br35H dataset to compare several CNN architectures for brain tumour detection: LeNet, AlexNet, VGG16, VGG19, and ResNet50. Among the models, AlexNet with the SGD optimiser performed the best, with good sensitivity/specificity metrics, a 98.82% F1-score, and an accuracy of 98.79%. These architectures are useful for classification, but they lack real-time inference and segmentation capabilities, which restricts their practical use in clinical settings.

On the other hand, our suggested YOLOv11-based system, which is optimised for real-time execution, carries out combined detection and segmentation. Our framework combines spatial localisation with boundary detection, which makes it more appropriate for extensive clinical workflows requiring tumour delineation and quantification, even if classification models such as EfficientNet-B4 are excellent for static tumour identification.

## Methodology

**Fig. 1:**
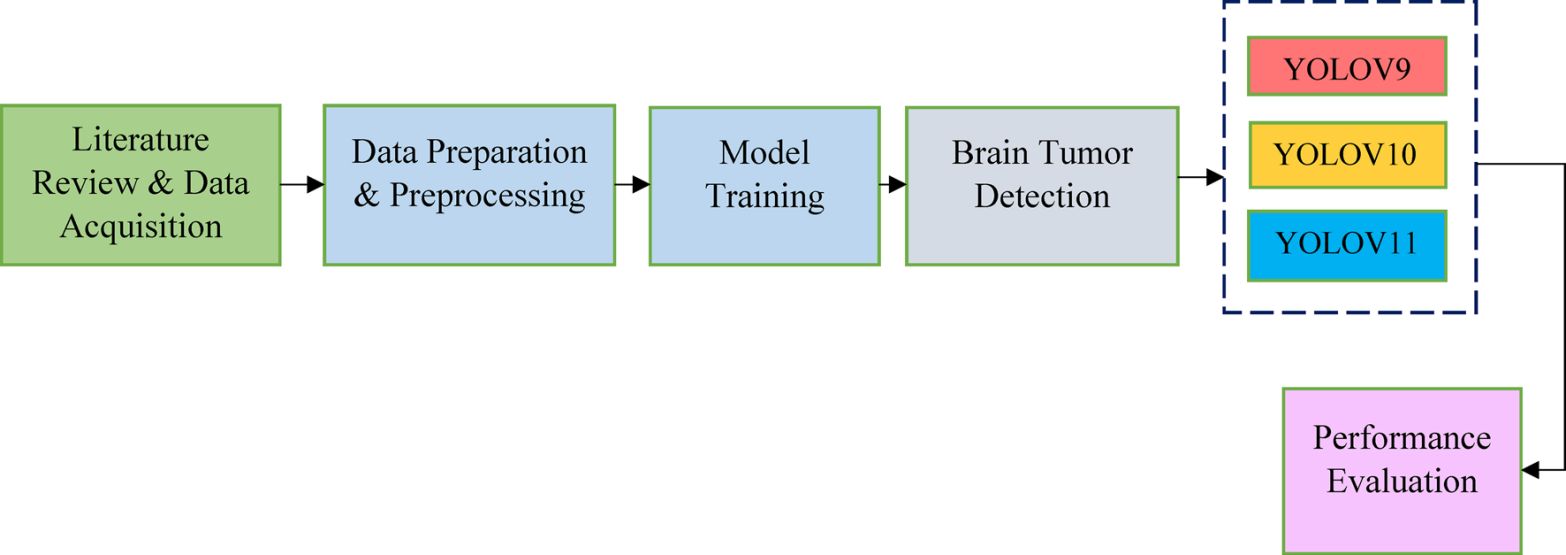
YOLO-based research flow diagram for brain tumour segmentation.

The main phases of the proposed research are outlined in Fig. 1, which begins with a comprehensive evaluation of the appropriate literature, including the most current studies, and culminates with the collection of datasets. The data then goes through selection and preparation followed by preprocessing. These procedures are used to train and assess the suggested model using standard parameters and hence its performance metrics evaluation.

**Fig. 2:**
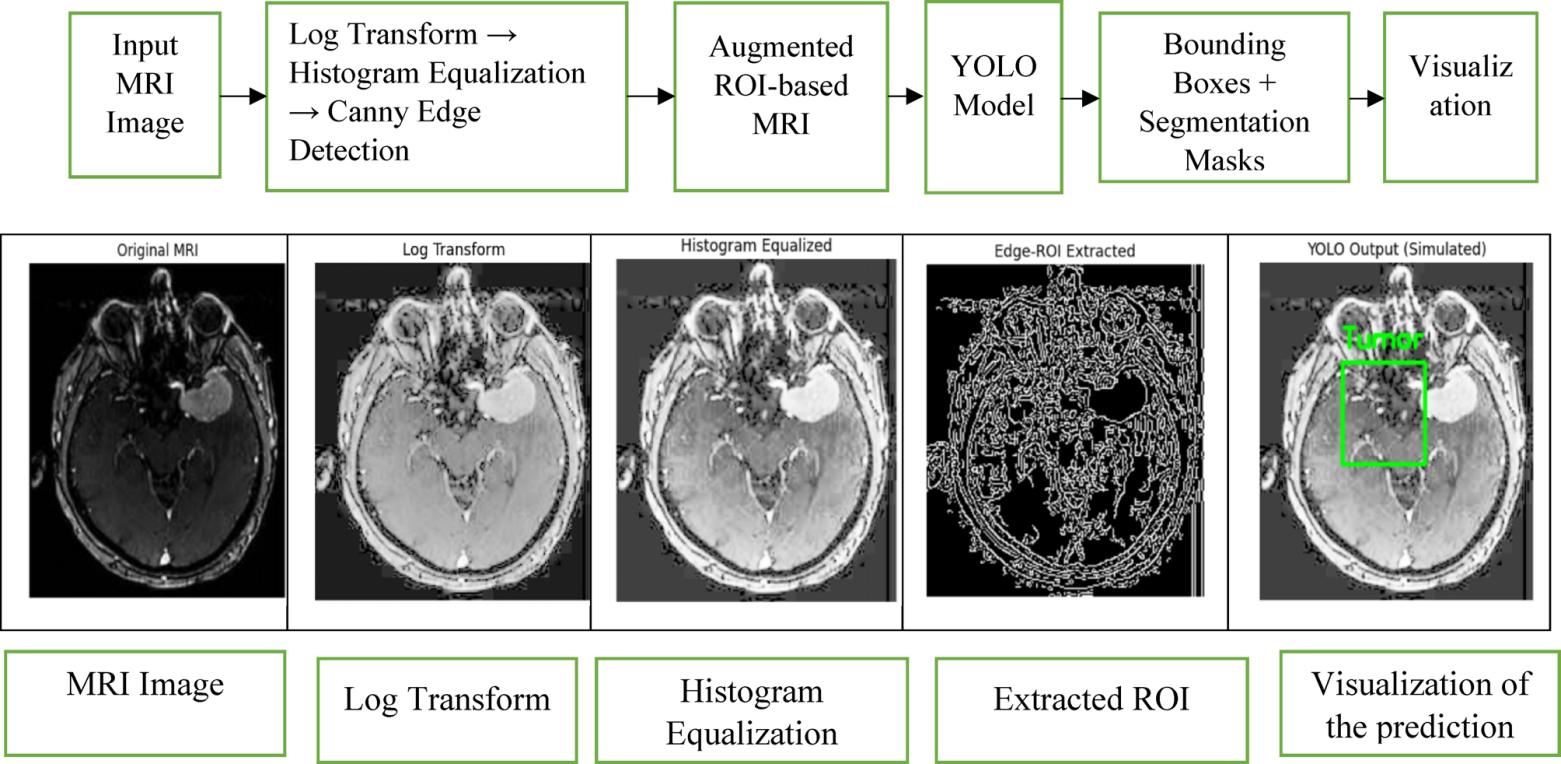
Proposed augmented MRI-based tumor detection pipeline with stage-wise output visualization.

The whole pipeline for MRI-based tumour identification utilising a YOLO model is depicted in the Fig. 2. For ROI enhancement, the procedure consists of log transformation, histogram equalisation, and Canny edge detection. The result from each step is displayed to highlight how preprocessing affects MRI pictures. Bounding box localisation and tumour area segmentation are included in the final forecast.

### Data collection

**Fig. 3:**
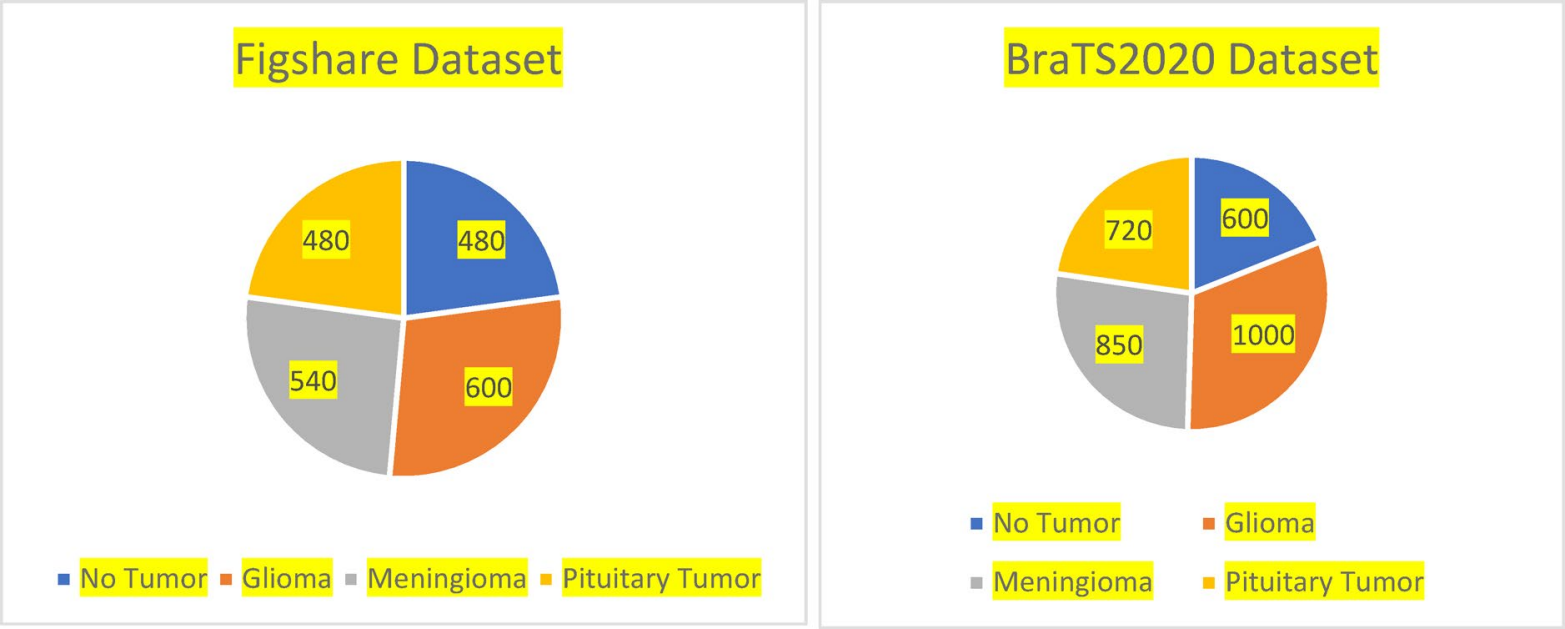
Dataset distribution across class.

**Fig. 4:**
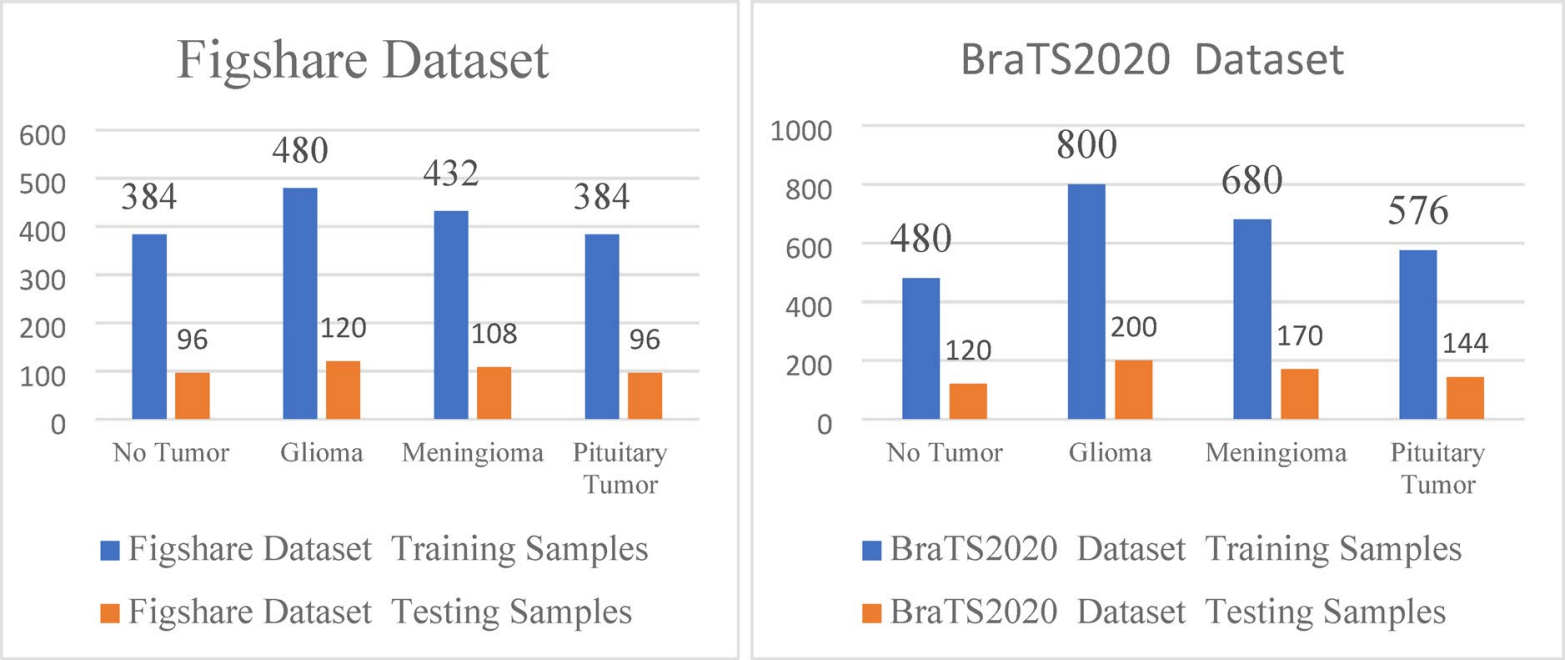
Dataset distribution for training and testing the model.

Figures 3 and 4, indicates that there are a total of 5270 photos, and was sourced from^56,66^. Each picture belongs to one of the following classifications: No Tumour, Glioma, Meningioma, or Pituitary Tumour. The relative proportions of these classes are 22.86%, 28.57%, 25.71%, and 22.86% in the Figshare dataset and 18.93%, 31.55%, 26.81%, and 22.71% in the BraTS2020 dataset. The No Tumour class indicates healthy brain scans, but the latter three classes together reflect tumor-positive patients, as seen in Fig. 1, 512 pixels make up each slice.

The class-wise distribution of samples in the BraTS2020 and Figshare datasets is shown in Fig. 3. In all datasets, gliomas make up the biggest fraction, followed by meningiomas and pituitary tumours; samples of no tumour are quite rare. In order to guarantee equitable model performance across all classes, the observable class imbalance emphasises the necessity of cautious handling throughout training.

To mitigate class imbalance, in addition to standard data augmentation namely rotation, flipping, scaling was employed class-weighted loss functions during model training. Oversampling was also applied in underrepresented classes within training batches to ensure balanced gradient updates.

**Fig. 5:**
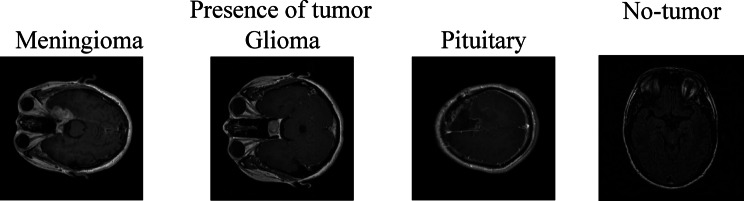
Sample MRI images from the dataset.

The distribution of training and testing across tumour classes for the BraTS2020 and Figshare datasets is displayed in Fig. 4. Even though the divides in the two datasets are comparable, the class-wise representation is unbalanced, particularly when there are more Glioma samples. Biassed learning and worse performance on minority classes might result from such unequal proportions across training and assessment sets. The visual representation of MRI in the absence of brain tumor and in the presence of brain tumor with grading levels are shown in Fig. 5.

### Pre-processing

The preprocessing pipeline accomplished three essential tasks by performing data augmentations while standardising pixel intensity values to the [0, 1] interval and adjusting image dimensions to 512 × 512 pixels. The automated contour refining procedure enables the mask alignment approach to solve this problem.

**Fig. 6:**
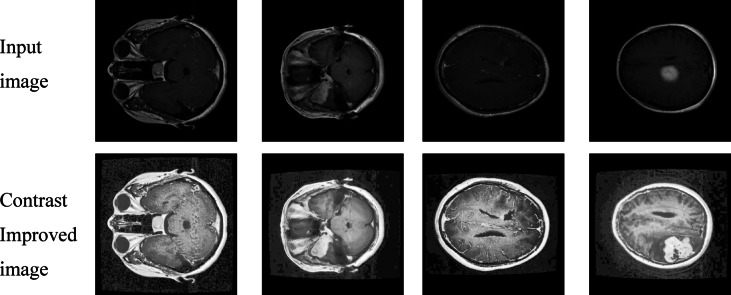
Enhanced brain MRI images using histogram equalization for improved contrast.

**Fig. 7:**
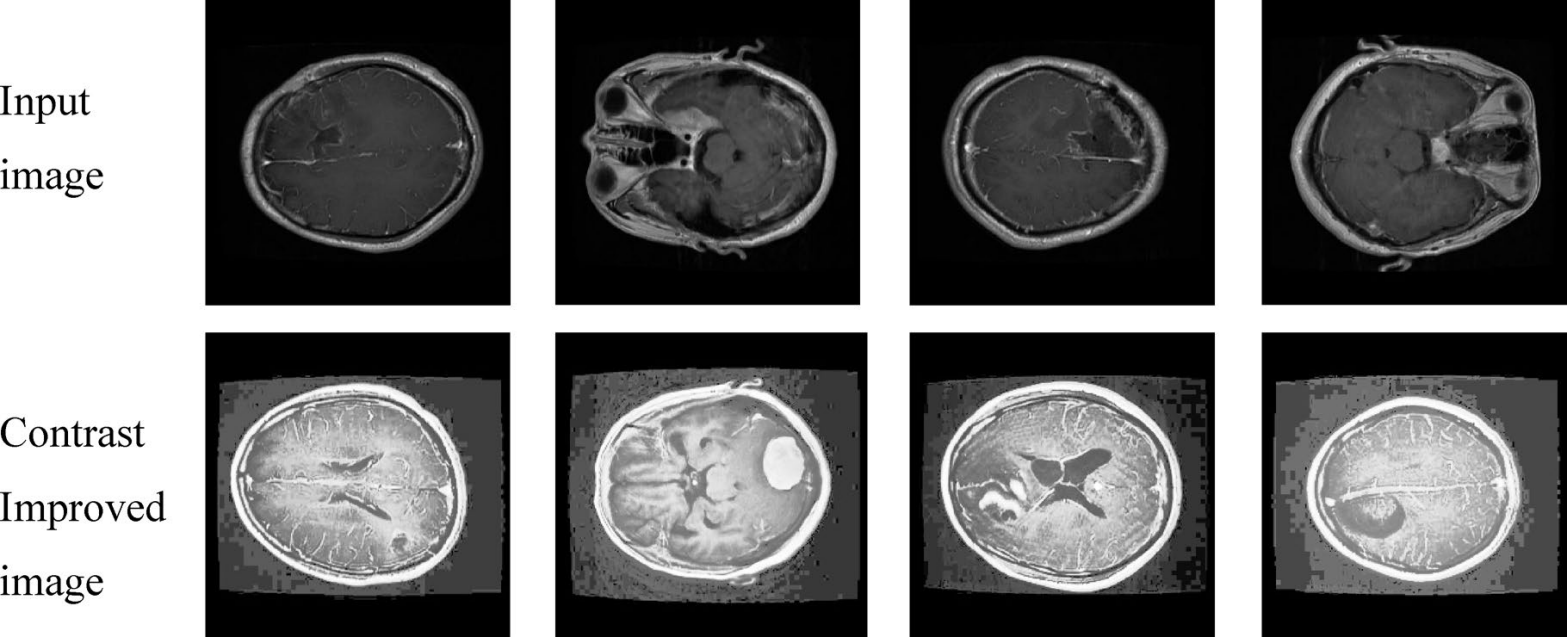
Enhanced brain MRI images using histogram equalization for improved contrast.

Using Histogram equalization technology enhances MRI brain scans by improving their contrast which makes tumor detection regions easier to observe shown in Figs. 6 and 7. The initial preprocessing operation makes it easier for the YOLO model to detect and segment brain tumors correctly.

**Fig. 8:**
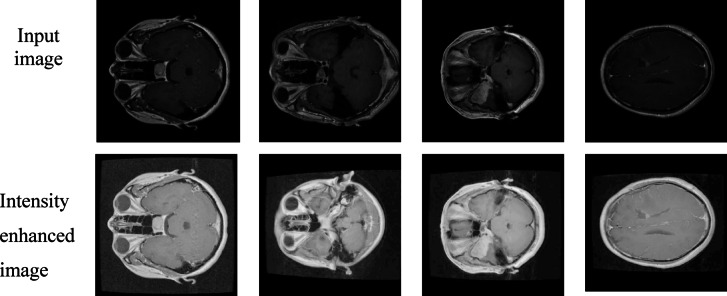
Brain MRI images after log transformation for intensity enhancement.

**Fig. 9:**
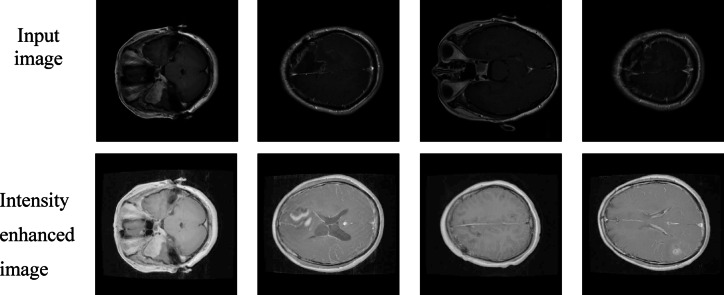
Brain MRI images after log transformation for intensity enhancement.

Log transformation is applied to MRI brain images to enhance low-intensity tumor regions by compressing high-intensity values shown in Figs. 8 and 9. This preprocessing step helps the YOLO model detect subtle tumor features more effectively.

A morphological dilation operation with a 3 × 3 kernel and 2 iterations was applied to ensure complete tumour coverage after OpenCV detected tumour edges in the original masks. These masks were then processed using a coordinate transformation method that linked images and their marked edges pixel-by-pixel, with tumour centroids serving as reference points.

Even while separate methods like log transformation and histogram equalisation are widely used, they provide a unique integration when combined with edge-based ROI extraction, which is especially suited for improving segmentation in YOLO-based models. Using these methods to provide segmentation-consistent input for detection tasks is the main novelty. As seen in Tables 4, 5 and 8, this alignment directly increased mAP@0.5 by 2.8% and recall by 1.2%, especially for complicated tumour types like gliomas.

**Fig. 10:**
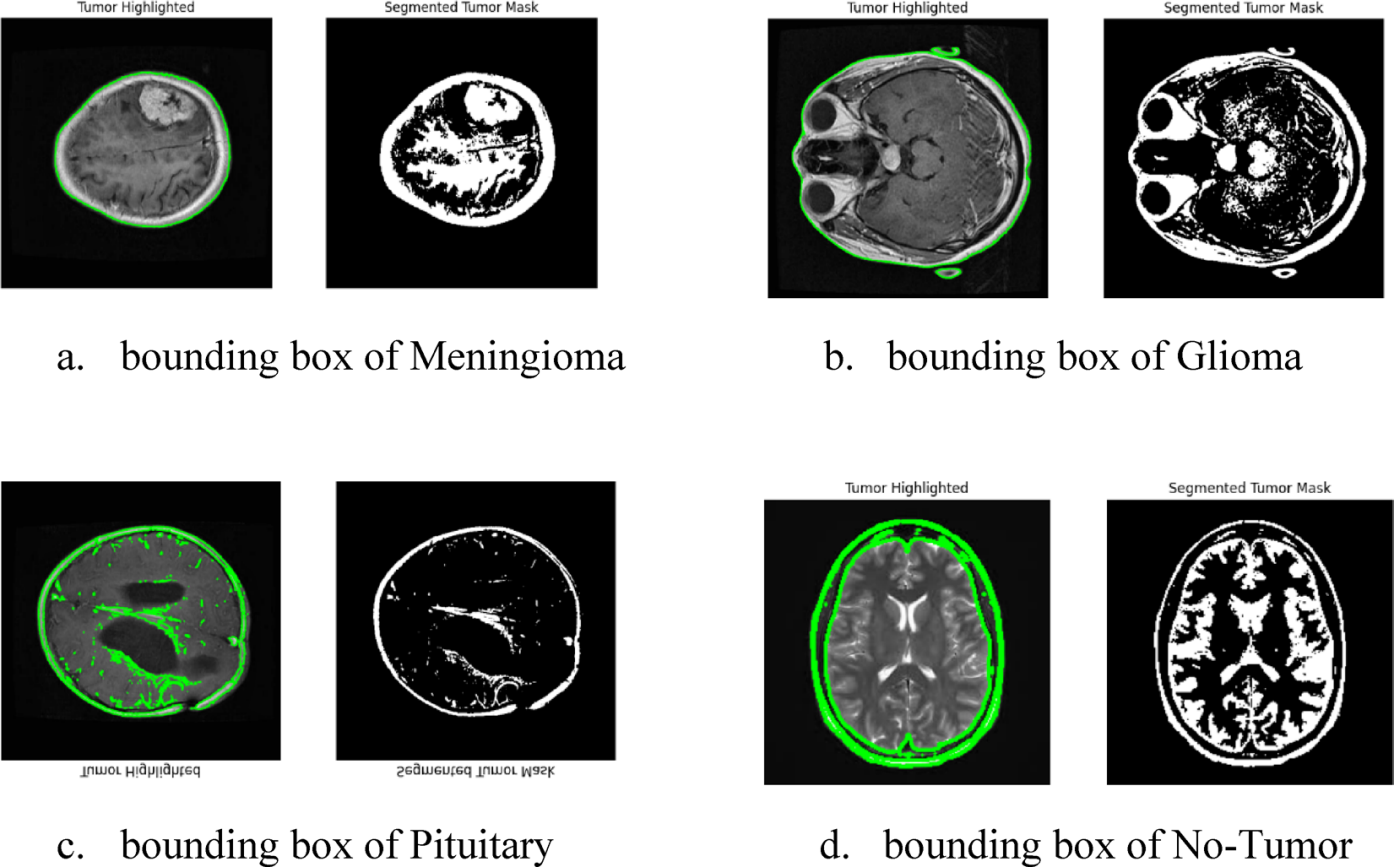
MRI images with bounding box and mask.

The impact of mask alignment on accuracy was evaluated by training YOLOv11 with 500 aligned and unaligned image examples. Table 7 shows the model achieved an mAP@0.5 of 0.965 without alignment and 0.993 with alignment. Recall results were 0.972 without alignment and 0.984 with alignment. Tumour localization accuracy improved by 2.8% mAP@0.5 and 1.2% recall, especially for irregularly shaped tumours like gliomas. These results show that aligned masks enabled more precise segmentation and tighter bounding boxes, allowing YOLOv11 to outperform its rivals in Fig. 10.

### Model training

Three variants of YOLO framework namely YOLOv9, YOLOv10 and YOLOv11 were used in this study.

(A) *YOLOv9 Model*.
  YOLOv9 is a DL framework that excels in object detection, segmentation, and classification. It improves on previous YOLO versions with advanced backbone structures, better spatial feature extraction, and an anchor-free detection process. YOLOv9 is well-suited for detecting brain tumours such as meningioma, glioma, and pituitary tumours in MRI scans.
  Feature extraction starts with Conv layers, and the C2f layers enhance gradient flow and representation learning. The SPPF module extracts features at different levels, while Transformer-based operations improve global contextual awareness. Adaptive up sampling and feature fusion layers refine tumour segmentation boundaries. Tumour masks and classification result from the combination of segmentation and classification. The model uses an anchor-free system for detection, improving versatility, with bounding box predictions consisting of five main parameters.
  t_x_,t_y_ : Centre offsets for localization.
  t_w_,t_h_ : Width and height of the detected tumor region.
  t_o_ : Confidence score for the presence of a tumor.

**Pseudo Code for Brain Tumor Segmentation - YOLOv9**.

*Step 1: Begin*.

*Step 2: Initialize YOLOv9 with a Transformer-based backbone*,* and integrate PANet and CSP modules for enhanced feature fusion*.

*Step 3: Acquire MRI input*.

*Step 4: Preprocess the input by resizing*,* normalization*,* and applying spatial augmentations*.

*Step 5: Extract features using multi-scale Transformer encoders to capture global contextual information*.

*Step 6: Iterate through each layer of the YOLOv9 architecture*:

*Step 7: If the current layer belongs to CSP modules*:

*Step 8: Implement Cross-Stage Partial connections to promote efficient information flow*.

*Step 9: Else if the layer is a Transformer encoder*:

*Step 10: Utilize self-attention mechanisms to model long-range dependencies*.

*Step 11: Else if the layer is part of the PANet structure*:

*Step 12: Merge multi-scale features using a path aggregation strategy*.

*Step 13: Else if the layer corresponds to the detection head*:

*Step 14: Execute object classification and bounding box regression through a decoupled head*.

*Step 15: End of conditional operations and loop*.

*Step 16: Apply Non-Maximum Suppression (NMS) to filter overlapping predictions and retain optimal bounding boxes*.

*Step 17: Generate final outputs: tumor bounding boxes*,* class confidence scores*,* and relevant feature representations*.

*Step 18: End*.

(B) *YOLOv10 Model*.

YOLOv10 is an advanced DL framework for real-time object detection, segmentation, and classification. It enhances previous YOLO versions with better feature extraction, attention mechanisms, and multi-scale processing, making it ideal for medical imaging tasks, including severity-based segmentation and classification.

The model uses Conv layers for low-level feature extraction, followed by C2f layers to improve feature learning. The Dynamic Spatial Pyramid Pooling (DSPP) module replaces traditional SPPF for better multi-scale feature fusion. It also employs adaptive up sampling and transformer-based attention mechanisms to refine tumour boundaries. The pipeline involves tumour mask generation with a segmentation head, tumour type classification with a classification head, and bounding box prediction for localisation.

**Pseudo Code for Brain Tumor Segmentation - YOLOv10**.

*Step 1: Begin*.

*Step 2: Set up YOLOv10 incorporating EfficientViT as the backbone and an anchor-free detection mechanism*.

*Step 3: Acquire MRI scan input—either from a static dataset or a live imaging source*.

*Step 4: Preprocess the image by resizing*,* normalizing intensity values*,* and applying light augmentations*.

*Step 5: Perform feature extraction using EfficientViT to ensure fast and efficient inference*.

*Step 6: Loop through each layer in the YOLOv10 architecture*:

*Step 7: If the layer belongs to the NAFBlock group*:

*Step 8: Implement nonlinear attention fusion for effective feature integration with minimal computational cost*.

*Step 9: Else if the layer is part of the Token Mixer group*:

*Step 10: Leverage token mixing to model global relationships across the image*.

*Step 11: Else if the layer is in the Anchor-Free Head*:

*Step 12: Predict object location*,* size*,* and category directly without relying on predefined anchor boxes*.

*Step 13: Else if the layer is from the Edge Refinement module*:

*Step 14: Enhance tumor boundary precision using adaptive smoothing techniques*.

*Step 15: End conditional checks and complete the loop*.

*Step 16: Apply a fast*,* dynamic non-maximum suppression technique to retain the best possible detections*.

*Step 17: Generate final outputs*,* including tumor bounding boxes*,* confidence levels*,* and a segmentation heatmap*.

*Step 18: End*.

(C) *YOLOv11 Model*.

YOLOv11 is an advanced DL framework designed for real-time segmentation and classification of brain tumors in MRI images. It excels in detecting and segmenting meningiomas, gliomas, and pituitary tumors with high accuracy, thanks to its optimized feature extraction and multi-scale attention mechanisms that enhance spatial feature fusion.

YOLOv11 was chosen for its hybrid backbone combining CNN, Vision Transformers (ViT), and sparse convolutional layers, allowing the model to effectively balance local detail capture and global contextual awareness. The integration of C2PSA modules enhances spatial focus on tumor regions, particularly in irregular and low-contrast areas. Additionally, Spatial Pyramid Feature Fusion (SPFF) blocks allow the model to aggregate multi-scale information for better segmentation accuracy. Sparse convolution was adopted to minimize redundant computation and improve inference speed.

The training hyperparameters were selected through empirical convergence analysis. A learning rate of 0.0001 offered stable loss behaviour, while momentum (0.9) and weight decay (0.0005) were used to optimize convergence and generalization. The model was trained for 50 epochs, which was determined to be optimal based on observed plateaus in validation loss and mAP performance metrics.

**Fig. 11:**
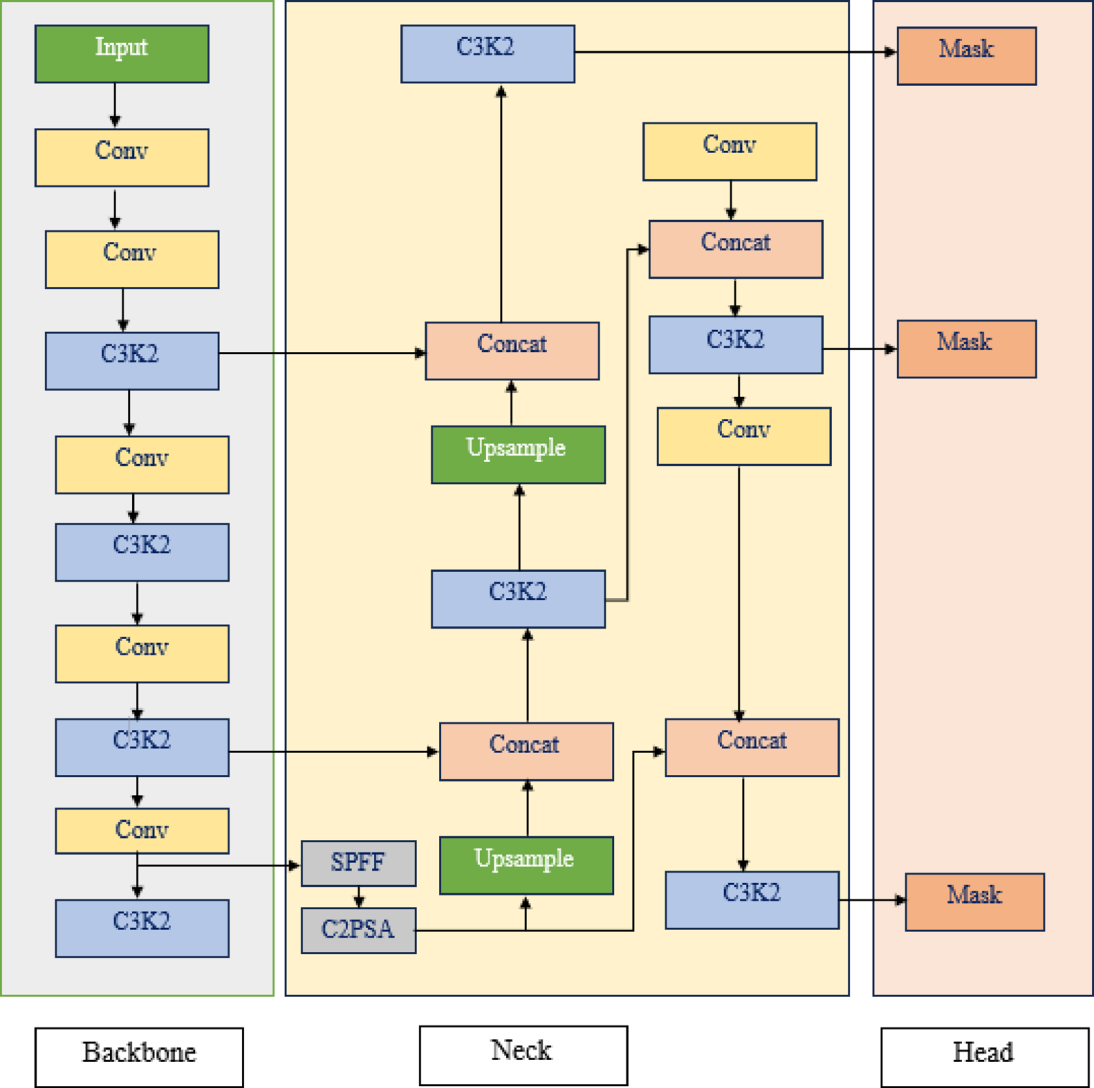
YOLOv11 architecture.

The architecture in Fig. 11, comprising the head, neck, and backbone, is crucial for tumor segmentation. Due to their irregular contours, varying sizes, and diverse textures, brain tumors are challenging to identify. The Backbone component of UUNET uses convolutional layers and C3K2 blocks to analyze MRI images and extract spatial features for detecting both detailed and large-scale tumor information. YOLOv11 applies C2PSA and SPFF attention mechanisms at the Neck layer for enhanced contextual understanding, performing up sampling and concatenations. This method improves the model’s adaptability to the various features present in brain tumors. Refined features are processed in the Head section, where segmentation masks are generated across different output scales.

**Pseudo Code for Brain Tumor Segmentation - YOLOv11**.

*Step 1: Begin*.

*Step 2: Initialize YOLOv11 with a hybrid backbone combining CNN*,* Transformer*,* and Vision Transformer (ViT) architectures*,* along with dynamic sparse convolution modules*.

*Step 3: Acquire the input image from a camera feed or an image source*.

*Step 4: Perform preprocessing by resizing the image*,* normalizing pixel intensities*,* and applying advanced data augmentations*.

*Step 5: Extract features using a combination of multi-scale CNN filters and Transformer-based embeddings*.

*Step 6: Iterate over each layer in the YOLOv11 model*:

*Step 7: If the layer belongs to the sparse convolution module*:

*Step 8: Execute sparse convolution with dynamic activation pruning for computational efficiency*.

*Step 9: Else if the layer is part of the attention module*:

*Step 10: Apply a hybrid attention mechanism integrating both CNN and ViT features*.

*Step 11: Else if the layer is involved in global feature fusion*:

*Step 12: Merge features across multiple scales and incorporate temporal information for context-aware detection*.

*Step 13: Else if the layer performs up sampling*:

*Step 14: Conduct adaptive up sampling to improve segmentation detail and spatial accuracy*.

*Step 15: Else if the layer is part of the segmentation module*:

*Step 16: Apply refined segmentation techniques focused on edge enhancement and accurate object boundary detection*.

*Step 17: End loop and conditional checks*.

*Step 18: Use a dynamic threshold-based non-maximum suppression method to filter overlapping detections*.

*Step 19: Generate final output comprising detected tumor regions*,* associated confidence scores*,* and predicted severity classification*.

*Step 20: End*.

### Performance evaluation

The metrics namely Recall, Precision, F-Score^59,67^ are measured and are discussed as follows.

1. *Recall* is measured by finding out the ratio between true positive (T_p_) to the sum of true positive (T_p_) and false negative (F_N_).
2. *Precision* is determined by dividing Tp by the sum of Tp and false positive (FP), it is used to specify the bounding box’s accuracy.
3. *F1-Confidence Curve*is the he graph that shows how the F1 score and the detecting application’s confidence threshold correlate.
4. *Confusion matrix*: This metric provides the information regarding precision and misclassifications of the proposed method. The matrix element is of the form (i, j) where i represents instance and j represents the prediction.
5. *Loss*: The loss function is used to minimise the loss in order to maximise the training performance of the model. Mathematically,
  1 $${\text{loss}}\,=\,{{\text{I}}_{{\text{box}}}}+{\text{ }}{{\text{I}}_{{\text{cls}}}}+{\text{ }}{{\text{I}}_{{\text{obj}}}}$$
  I_box_ is the regression of bounding box, I_cls_ is the loss due to classification and I_obj_ is the loss due to confidence.
  2 $$\begin{aligned}\:{\text{I}}_{\text{b}\text{o}\text{x}}\hspace{0.17em}&=\hspace{0.17em}{}_{\text{c}\text{o}\text{o}\text{r}\text{d}}\:=\sum_{i=0}^{{s}^{2}}\sum_{j=0}^{B}{I}_{i,j}^{obj}bj(2-{w}_{i}*{h}_{i}\left)*\right[\:({x}_{i}-{x}_{i}^{j}){.}^{2}+({y}_{i}-{y}_{i}^{j}){.}^{2}\\ & \quad \:({x}_{i}-{x}_{i}^{j}){.}^{2}+\:({y}_{i}-{y}_{i}^{j}){.}^{2}+({w}_{i}-{w}_{i}^{j}){.}^{2}\:+\:({h}_{i}-{h}_{i}^{j}){.}^{2}\end{aligned}$$
  λ_coord_ is the positional loss’s weight, the central co-ordinates are denoted by x and y values, the target width and height is denoted by the variables w and h.
  3 $$\:{\text{I}}_{cls}\hspace{0.17em}=\hspace{0.17em}{}_{\text{c}\text{l}\text{s}}\:\sum_{i=0}^{{s}^{2}}\sum_{j=0}^{B}\sum_{classes}{p}_{i}\left(c\right)\text{l}\text{o}\text{g}(\text{P}\text{i}`\:(\text{c}\left)\:\right)$$
  λ_cls_ is the category loss, P_i_(c) is the target’s probability which belongs to a class, P_i_$\:`$ (c) is the value of actual class.
  4 $$\:{\text{I}}_{obj}\hspace{0.17em}=\hspace{0.17em}{{\uplambda\:}}_{\text{noobj}}\sum_{i=0}^{{s}^{2}}\sum_{j=0}^{B}{I}_{i,j}^{\text{noobj}}({c}_{i}-{c}_{i}^{\prime }){.}^{2}+\hspace{0.17em}{{\uplambda\:}}_{\text{obj}}\:\sum_{i=0}^{{s}^{2}}\sum_{j=0}^{B}{I}_{i,j}^{obj}({c}_{i}-{c}_{i}^{\prime }){.}^{2}$$
6. *Mean Average Precision*: mAP @0.5 represents the mean Average Precision at intersection over union (IoU) threshold of 0.5, mAP@0.5:0.95 refers to mAP calculated across various IoU thresholds from 0.5 to 0.95. It can be calculated as.
  5 $$\:\text{m}\text{A}\text{P}\:=\frac{1}{n}\sum_{n=1}^{k=n}{AP}_{k}$$
  n represents the classes and $\:{AP}_{k}$ represents the class k’s average value of precision.

All three YOLO models were trained for 50 epochs using SGD at a learning rate of 0.0001, optimized for generalization and convergence speed on the BT dataset. The learning rate of 0.0001 was selected over 0.00001 and 0.001 due to slow convergence at 0.00001 and unstable loss behaviour at 0.001. Validation loss was tracked in ten-epoch steps from 30 to 100 epochs, with all models plateauing at 50 epochs. For YOLOv11, validation box loss decreased from 0.82 at 30 epochs to 0.77 at 50 epochs, with minimal change at 60 epochs. The 50-epoch setting offered a balance between performance and training time. Momentum was set to 0.9, and weight decay to 0.0005 to improve gradient stability and prevent overfitting. Results from Section IV were based on iterative parameter adjustments using loss curves and mAP@0.5 scores, despite limitations in grid search due to computational constraints.

YOLOv9, YOLOv10, and YOLOv11 were selected for their progressive architectural improvements over earlier variants and traditional two-stage methods like R-CNN. YOLOv9 introduced the GELAN backbone for efficient feature propagation, YOLOv10 enhanced spatial context via SPPCSPCS and ERGELAN modules, while YOLOv11 incorporated sparse convolution and attention modules (C2f + ViT + SPFF) to better detect irregular tumor shapes with low contrast. These variants balance real-time detection with high segmentation accuracy, making them ideal for medical imaging tasks.

A comparison of the YOLOv9, YOLOv10, and YOLOv11 designs designed for segmentation and hence severity grading of brain tumour is shown in the Table 2.

**Table 2:** YOLO models architectural comparison.

Feature	YOLOv9	YOLOv10	YOLOv11
Backbone	Transformer-based	EfficientViT	Hybrid CNN + Transformer + ViT
Segmentation support	Bounding box only	Bounding box + heatmap	Full segmentation with mask and edge refinement
Attention	Global self-attention	Token mixer (lightweight)	Hybrid attention (CNN + ViT)
Detection head	Anchor-based, decoupled	Anchor-free	Multi-scale detection with severity prediction
Efficiency	Moderate	High (real-time capable)	High with sparse computation

## Results and discussion

All the observed results using the YOLO models are included in this section. Recent advancements in brain tumor detection have demonstrated the efficacy of hybrid techniques^68^, label-free synthesis^43^, and robust classification models^44,45^. Our proposed YOLOv11 framework aligns with these trends, offering real-time performance while maintaining high accuracy. Novel detection methods, such as those proposed by^46^, and multimodal segmentation approaches^47^, further underscore the importance of integrating diverse imaging modalities for improved diagnostic outcomes.

### Experimental setup

Google Colab Pro + provided robust cloud-based resources for model training and evaluation, utilizing a 2.2 GHz Intel Xeon CPU, 64 GB RAM, and a V100 GPU with 128 GB VRAM. YOLOv10 and YOLOv11 were optimized for these resources, while YOLOv9, with 27.63 M parameters and 157.6 GFLOPs (Table 3), needed additional resources. Training used 16 batches, limited by GPU RAM. Data loading was done via PyTorch’s Dataloader with four worker threads. The test in “Detection time” ensured hardware consistency. YOLOv11 took 0.257 h, YOLOv10 took 0.296 h, and YOLOv9 took 0.608 h. Post-processing and inference were conducted on the same GPU with a single-threaded Python script. Repositories were moved to Google Drive.

**Table 3:** Computational and architectural comparison of YOLO models.

Metric	YOLOv11	YOLOv10	YOLOv9
Layers	113	129	169
Parameters	2.83 M	8.04 M	27.63 M
GFLOPs	10.2	24.4	157.6

YOLOv11, with 2.83 M parameters and 113 layers, is ideal for real-time tumour screening and portable scanners. YOLOv10, with 8.04 M parameters and 129 layers, balances speed and accuracy, making it suitable for clinical diagnoses with strict efficiency requirements. YOLOv9, with 27.63 M parameters, excels in accuracy but needs high computational resources, shown by its 157.6 GFLOP capacity. YOLOv10 delivers 24.4 GFLOPs, while YOLOv11 achieves 10.2 GFLOPs. Model selection depends on whether precision, system performance, or adaptive screening is prioritized.

We used the SGD optimizer with momentum (0.9), weight decay (0.0005), and an initial learning rate of 0.0001. These values were chosen based on validation loss stability and convergence speed observed in earlier training runs. A batch size of 32 and a maximum of 50 epochs provided the best generalization, as performance plateaued beyond this point.

### Performance metrics

i. *Precision & Recall*.
  The evaluated precision and recall values of YOLO models on the said dataset is visualised in Tables 4 and 5.
  Table 3 displays the precision values obtained by YOLOv9, YOLOv10 and YOLOv11 when detecting tumors in two classes namely Tumor Positive containing 306 images and Tumor Negative containing 75 images. The class-wise performance of the YOLOv11 model, with and without augmentation strategies, is presented in Table 6.
  Top precision results indicate that YOLOv11 reaches 0.9921 for Tumor Positive detection while achieving 0.9916 for Tumor Negative detection even higher than YOLOv9’s 0.992 and 0.99 and YOLOv10’s 0.9905 and 0.9898.
  The recall data for YOLO models are presented in Table 4. YOLOv11 leads the group of three models with 0.9918 recall followed simultaneously by YOLOv10 at 0.9915 and YOLOv9 at 0.988. The experimental data shows that YOLOv11 stands as the top performer while displaying a slight superiority in both the classes.
  The effect of augmentation techniques on the YOLOv11 model’s performance is shown in Fig. 8, where recall and precision values for tumor-classes are displayed. The findings unequivocally demonstrate that using augmentation approaches enhanced precision from 0.984 to 0.992 and recall from 0.983 to 0.9921 for both classes. These improvements show that the techniques used successfully decreased class bias and improved the model’s capacity to accurately detect and differentiate between tumour types.
  We used 5-fold cross-validation across the combined Figshare and BraTS datasets to guarantee robustness and reduce the impact of dataset-specific biases. Five equal portions of the dataset were randomly selected; four of the folds were used for training and one as the test set. Accuracy, precision, recall, F1-score, and mAP@0.5 were the final performance parameters that were averaged over the five folds shown in Table 7. This approach decreased the danger of overfitting and offered a trustworthy estimate of model generalisation.
ii. Loss performance.
  Tables 8 and 9 compares the performance of YOLOv9, YOLOv10, and YOLOv11 models for brain tumor detection based on losses during training and validation, which includes loss in box, segmentation, classification, and Distribution Focal Loss (DFL).

**Table 4:** YOLO models performance based on precision values.

		Precision values		
Class name	Total images	YOLOv9	YOLOv10	**YOLOv11**
Tumor positive	306	0.992	0.9905	**0.9921**
Tumor negative	75	0.99	0.9898	**0.9916**

Significant values are in bold.

**Table 5:** Performance of YOLO models based on recall values.

		Recall		
Class name	Total images	YOLOv9	YOLOv10	**YOLOv11**
Tumor positive	306	0.989	0.9921	**0.9921**
Tumor negative	75	0.988	0.9915	**0.9918**

Significant values are in bold.

**Table 6:** Class-wise recall and precision values of YOLOv11 model with and without augmentation strategies.

Class name	Without augmentation	With augmentation
***(a) Recall values***		
*YOLOv11-precision values*		
Tumor positive	0.984	**0.992**
Tumor negative	0.984	**0.992**
***(b) Precision values***		
*YOLOv11-recall values*		
Tumor positive	0.983	**0.9921**
Tumor negative	0.983	**0.9918**

Significant values are in bold.

**Table 7:** Average performance metrics across 5-fold cross-validation.

Model	Accuracy (%)	F1-Score	mAP@0.5	mAP@0.5:0.95	Std Dev (F1)
YOLOv9	96.1	0.958	0.994	0.802	0.004
YOLOv10	97.4	0.972	0.987	0.792	0.003
**YOLOv11**	**99.2**	**0.99**	**0.993**	**0.801**	**0.002**

Significant values are in bold.

**Table 8:** Training loss comparison of YOLO models.

Model	train/box_ loss	train/seg_ loss	train/cls_ loss	train/dfl_ loss
YOLOv9	0.72	1.19	0.42	1.28
YOLOv10	1.34	NA	0.68	2.01
**YOLOv11**	**0.61**	**1.04**	**0.32**	**0.96**

Significant values are in bold.

YOLOv11 demonstrates the most minimal loss values across all categories attributing to its better optimized learning capabilities. Meetings of object detection fill the lowest values of DFL (0.96), box (0.61), and classification (0.32) loss while demonstrating superior recovery ability and box precision and localization.

**Table 9:** Validation loss comparison of YOLO models.

Model	val/box_ loss	val/seg_ loss	val/cls_ loss	val/dfl_ loss
YOLOv9	0.76	1.28	0.39	1.31
YOLOv10	1.52	NA	0.7	2.04
**YOLOv11**	**0.77**	**1.24**	**0.33**	**1.02**

Significant values are in bold.

YOLOv11 demonstrates excellent generalization due to its competitive loss values which include 0.33 which is a classification loss and 1.02 which is DFL loss.

**Fig. 12:**
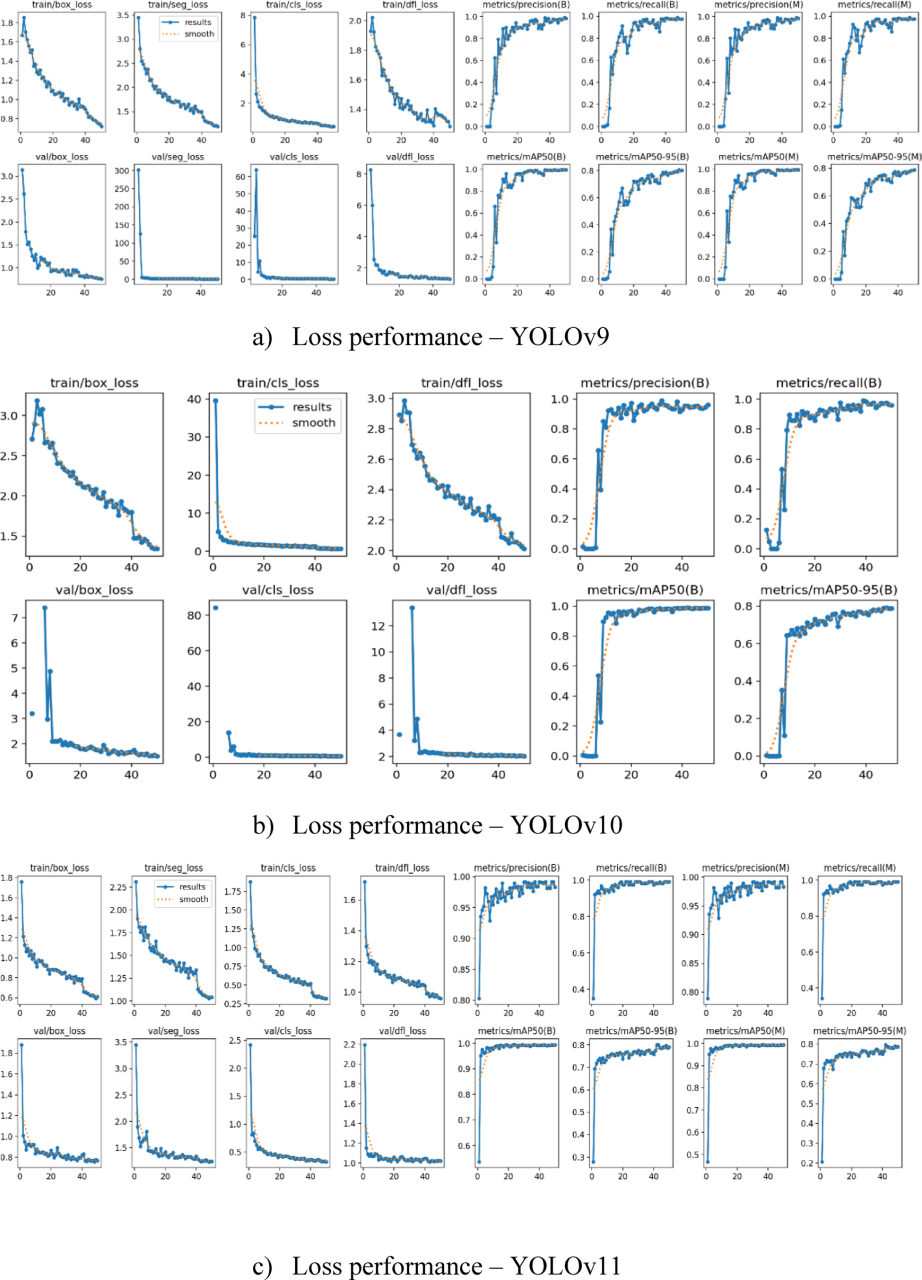
Loss performance of Yolo models in classifying brain tumor.

The brain tumour identification analysis in Fig. 12 confirms that YOLOv11 maintains superior outcomes than YOLOv9 and YOLOv10 across various performance parameters during training and validation steps. YOLOv11 achieves superior learning efficiency in tumor detection through its minimal loss measurements across all performance metrics including losses. YOLOv9 follows behind YOLOv11 with competitive performance although its loss totals surpass those of YOLOv11.

**Fig. 13:**
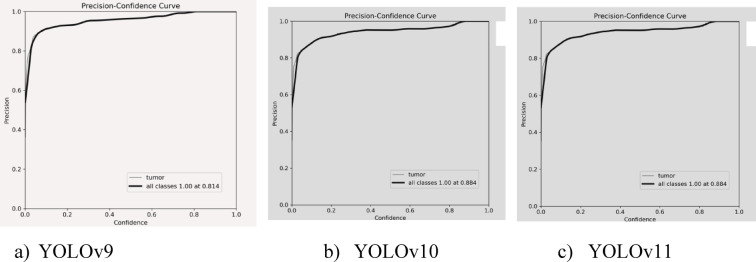
Precision Confidence curves of YOLO models.

Figure 13 shows through precision-confidence curves that all models continue delivering high accuracy results across various confidence levels for brain tumour testing. YOLOv9 achieves a precision of 0.814 that is slightly lower compared to YOLOv10 and YOLOv11 which reach 0.884 overall precision. Different aspects of YOLOv11 analysis show a balanced approach due to its enhanced tracking accuracy and higher recall rate in addition to reduced loss values.

**Fig. 14:**
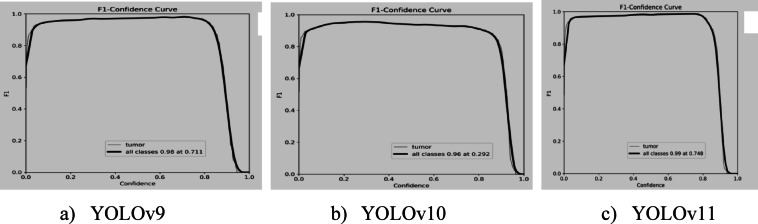
F1-confidence curves of YOLO models.

Brain tumour detection precision varies by balancing recall and accuracy, as shown in Fig. 14. YOLOv9 achieves its highest performance of 0.98 at 0.711 confidence, YOLOv11 reaches 0.99 at 0.748 confidence, and YOLOv10 shows 0.96 at 0.292 confidence. YOLOv11 consistently delivers near-perfect F1-scores at higher confidence levels, making it reliable for tumour segmentation tasks.

**Fig. 15:**
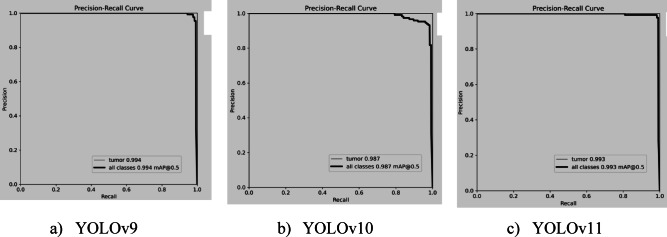
Precision Recall curves of YOLO models.

YOLOv9 achieves the highest accuracy with a mAP@0.5 of 0.994, minimizing false positives and demonstrating strong object detection. YOLOv11 follows closely with a mAP@0.5 of 0.993, while YOLOv10 lags slightly behind. Both YOLOv9 and YOLOv11 offer reliable precision-recall trade-offs, excelling in brain tumour segmentation. YOLOv11 stands out as the best practical choice for tumour detection, balancing efficiency with high accuracy and recall. Figure 15 illustrates the Precision-Recall curves of YOLO models.

YOLOv11 achieved the highest area under both ROC and PR curves, indicating its superior ability to maintain a high true positive rate while minimizing false positives.

### Qualitative analysis of tumor detection using YOLO models

**Fig. 16:**
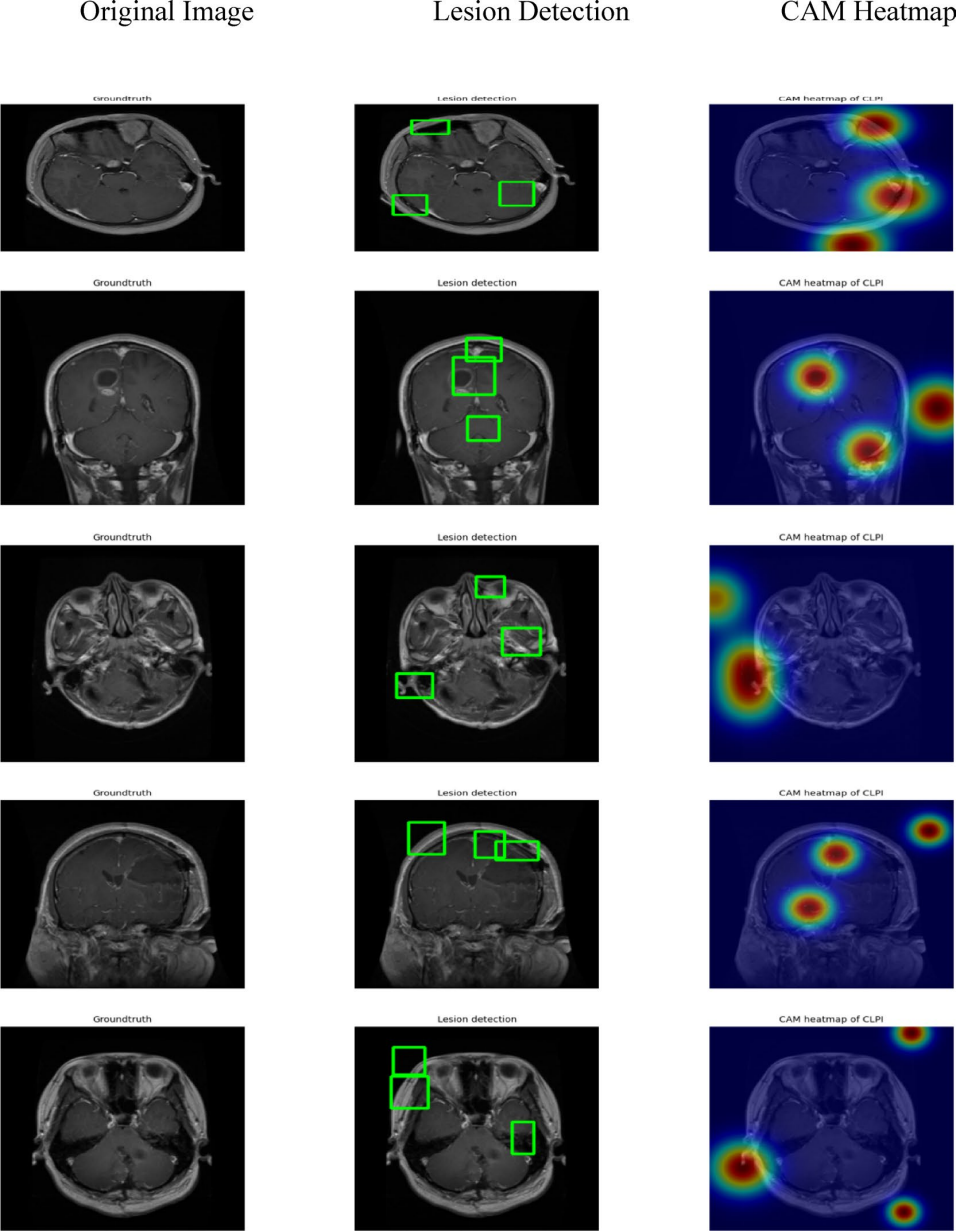

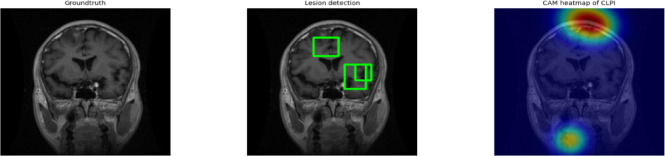
Lesion Localization and Interpretability using CAM.

Figure 16 presents qualitative results of tumour segmentation through three YOLO versions: YOLOv9, YOLOv10 and YOLOv11. The images in the detection performance section of each model display bounding boxes that should mark MRI data.

From Fig. 16 The first image shows the ground truth MRI scan with visible tumor regions. The second image highlights the lesion regions accurately detected by YOLOv11, indicated with green bounding boxes that closely align with the actual tumor locations. The third image presents a Class Activation Map (CAM) using CLPI, which confirms that the model focuses on clinically relevant areas during prediction, enhancing trust in its decisions.

**Fig. 17:**
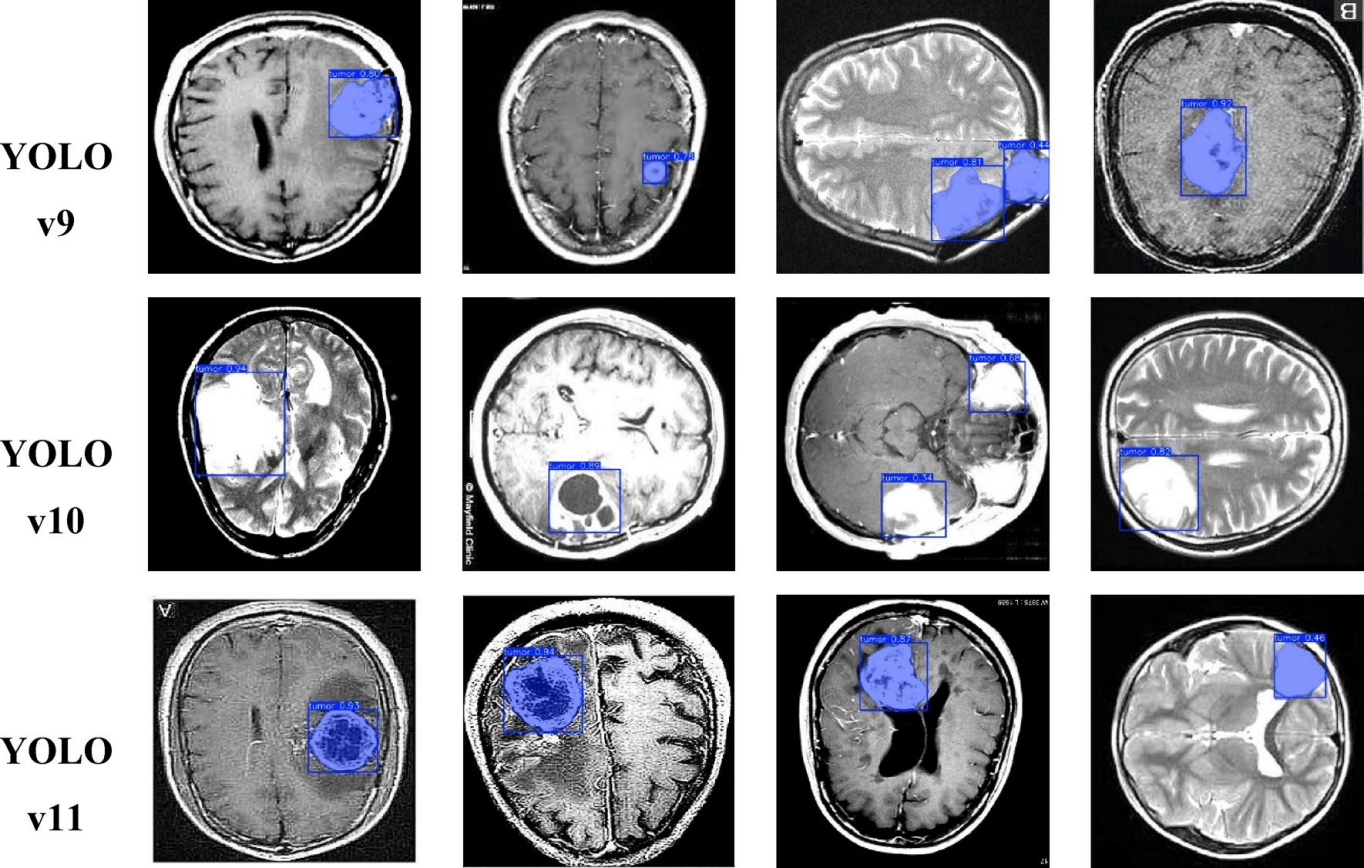
Visualization of tumor segmentation on MRI images using YOLO models.

**Fig. 18:**
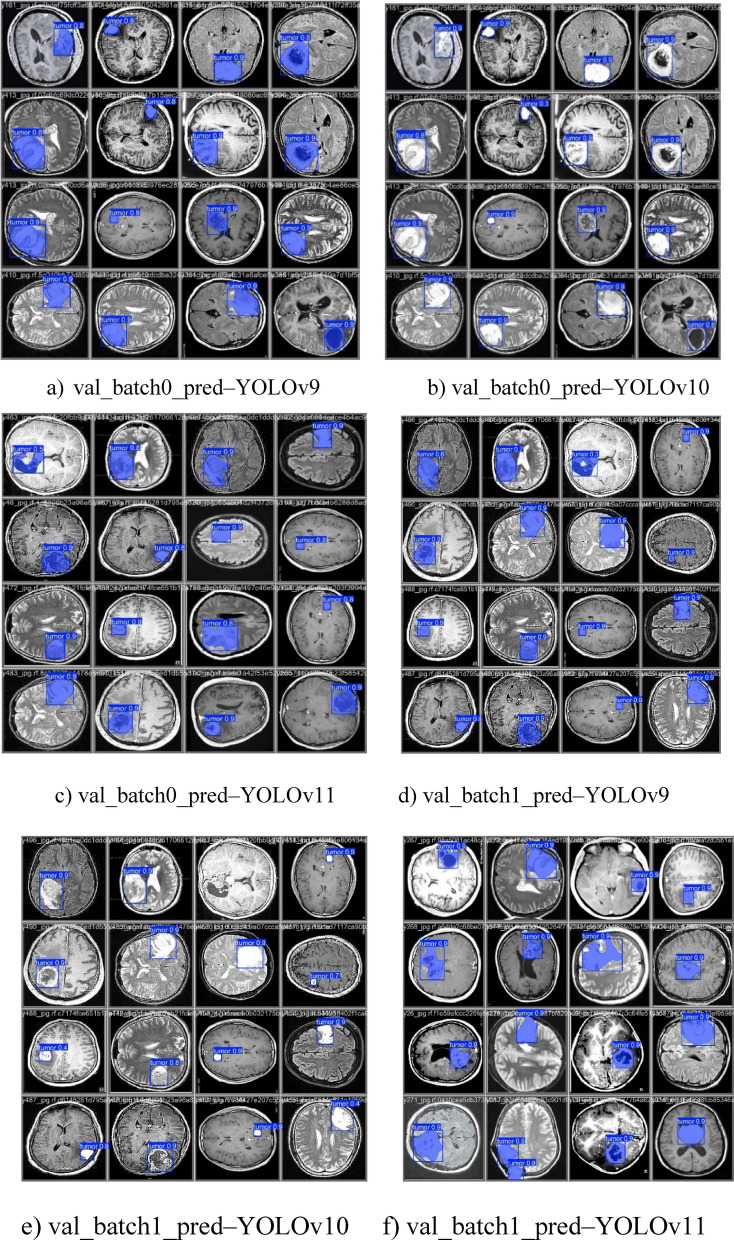
Visualization of tumor detection on MRI images using YOLO models.

Figures 17 and 18a–f shows validation batch predictions, where all models correctly locate tumour areas in MRI scans, with tumour boundaries marked by blue boxes. YOLOv9 and YOLOv11 fit bounding boxes well, while YOLOv10 shows some irregularities, potentially causing incorrect detections. YOLOv11 maintains stable high-confidence scores, achieving the highest accuracy in tumour localization, followed by YOLOv9. YOLOv10, while satisfactory, shows slightly lower detection precision.

### Confusion matrix

**Fig. 19:**
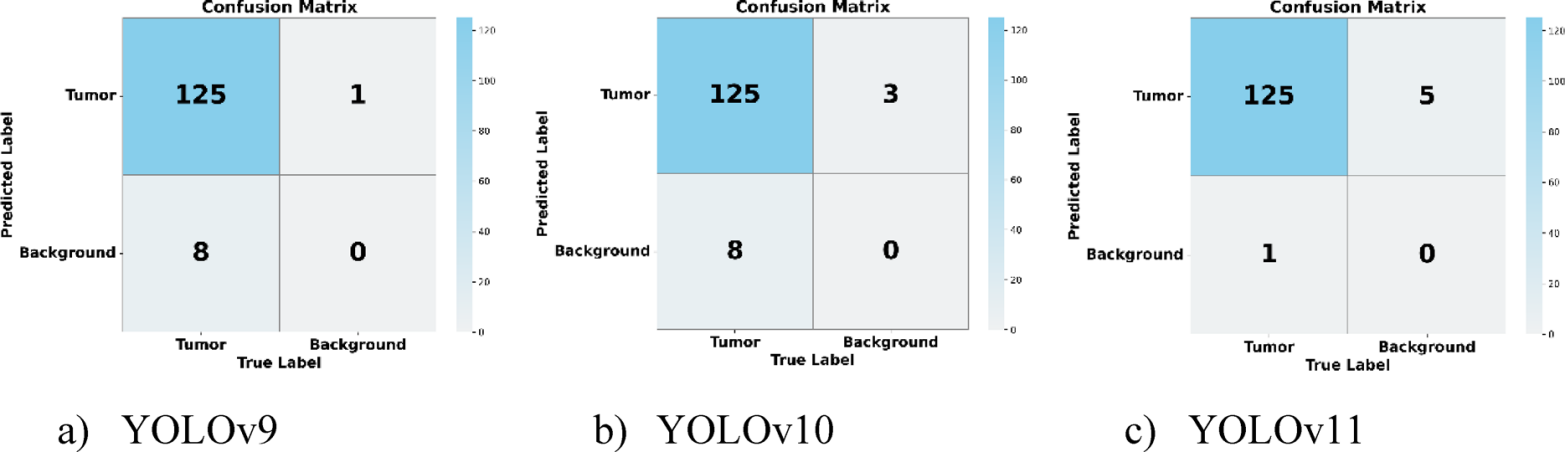
Confusion matrix of YOLO models.

Figure 19 demonstrates the way YOLO models perform in brain tumour segmentation. With high true positive values for tumour detection, all three models exhibit significant classification abilities.

The YOLOv9 and YOLOv10 models wrongfully label eight background specimens as tumours while YOLOv10 misidentifies three tumors as background examples. The false positive detection rate of YOLOv11 stands superior to its prior versions due to its 5 incorrectly labeled background samples. The results show that YOLOv11 effectively decreases false positives which proves its reliability in brain tumour segmentation.

### Detection time

The evaluation of the YOLO models combines inference speed, post-processing time, and training duration. YOLOv11 leads with 5.3 ms inference time, surpassing YOLOv10 (8.2 ms) and YOLOv9 (23.5 ms). However, YOLOv11’s post-processing is slower (8.5 ms) than YOLOv10 (0.4 ms) and YOLOv9 (3.4 ms). YOLOv11 also requires the least training time (0.257 h), followed by YOLOv10 (0.296 h) and YOLOv9 (0.608 h). These results emphasize YOLOv11’s superior speed and efficiency for speed-sensitive brain tumour detection tasks.

Table 10 compares the performance of YOLO models in brain tumour identification using important detection metrics for both segmentation mask (Mask) and bounding box (Box).

**Table 10:** Quantitative comparison of YOLO models.

Metrics	YOLOv9		YOLOv10		YOLOv11	
	Box	Mask	Box	Mask	Box	Mask
Precision (P)	0.984	0.984	0.938	0.93	**0.99**	**0.99**
Recall (R)	0.976	0.976	0.976	0.97	**0.984**	**0.984**
mAP50	**0.994**	0.994	0.987	0.97	0.993	**0.993**
mAP50-95	0.802	0.789	0.792	0.81	**0.801**	**0.796**

Significant values are in bold.

YOLOv11 achieves the highest performance with 0.99 accuracy and 0.984 recall, showcasing improved brain tumour detection through both bounding boxes and segmentation masks. YOLOv9 follows closely with 0.984 accuracy and 0.976 recall. Although YOLOv10 reaches a recall of 0.976, its accuracy is lower at 0.938, indicating more false positive detections. YOLOv9 leads in mAP@0.5 with 0.994, while YOLOv11 closely follows with 0.993, demonstrating comparable precision in tumour detection.

To verify whether the observed improvements in YOLOv11’s performance were statistically significant, we conducted paired t-tests on mAP and F1-score values from the test set across multiple runs. The p-values for comparisons between YOLOv11 and YOLOv10 were < 0.01 for both metrics, confirming that YOLOv11’s improvements are statistically significant and not due to random variation.

### Trade-offs and clinical implications

YOLOv9 achieves a 0.994 mAP@0.5 but operates with a 23.5 ms inference time, making it ideal for precise preoperative planning. YOLOv11, on the other hand, offers real-time tumour diagnosis in emergencies with high precision (0.99) at 5.3 ms. YOLOv10, with an 8.2 ms inference time, serves as an initial detection system in resource-constrained settings, without requiring segmentation. Clinical practice relies on these trade-offs to choose the optimal model, balancing resource efficiency with accurate identification at the necessary pace.

## Comparative analysis of segmentation models

Many currently used segmentation models demonstrate their performance metrics through Table 11 which employs a standardized dataset.

**Table 11:** Performance metrics of existing and the proposed models.

Algorithm	Precision in %	Recall in %	mAP@0.5 in %	Map@0.5:0.95 in %
RCNN^69^	x	95	91	x
Faster RCNN^69^	x	94	94	x
Mask RCNN^69^	x	95	94.8	x
YOLOv5	x	90.6	94.7	65.7
YOLOv7	x	90.3	94.1	65.9
**YOLOv9 [proposed]**	**99.1**	**98.85**	**99.4**	**80.2**
**YOLOv10[proposed]**	**99.015**	**99.18**	**98.7**	**79.2**
**YOLOv11[proposed]**	**99.185**	**99.195**	**99.3**	**80.1**

Significant values are in bold.

RCNN and its successors show detection recall rates of 94–95%, confirming their tumour detection ability. However, an undisclosed mAP score limit may affect precision. YOLO-based models, with their computational efficiency and accuracy, have notable advantages. YOLOv9, YOLOv10, and YOLOv11 models outperform existing models, achieving precision above 99% and recall up to 99.2%, ensuring highly accurate brain tumour segmentation. Their mAP@0.5 values exceed 98%, with YOLOv9 improving by 80.2%, YOLOv10 by 79.2%, and YOLOv11 showing 80.1% mAP@0.5:0.95, highlighting their robustness for detecting tumours of various sizes and shapes^70^. Table 12 presents the accuracy of different segmentation approaches for brain tumour imaging datasets.

**Table 12:** Performance of existing and proposed image segmentation methods across datasets.

Method	Database	Accuracy in %
Neural network^71^	Various images of Brain	93.03
Deep wavelet autoencoder^72^	RIDER	96
Combining noise-to-image and image-to-image GANs^73^	BraTS2016	91.01
Multimodal fusion network^74^	MICCAI BraTS2018	91.02
Convolutional neural network^75^	BraTS	98
Learning based system^76^	Meningioma, Pituitary, Glioma	86.01
LSTM based learning model^77^	BraTS2012 to BraTS2018	98
K-means and improved fuzzy C means clustering algorithm (template-based)^78^	40 MRI Brain Images	98
Depth first search tree segmentation^79^	MIAS	99
Intensity adjustment^80^	ANBU Hospital in Madurai database	97
CNN in MRI images^81^	BraTS2015	92
Concatenation approach^82^	3064 T1 - weighted contrast MR images	99
Extreme learning^83^	BraTS2012 to BraTS2015	95.01
Deep learning approach^84^	BraTS	95.01
**YOLOv9 [proposed]**	**BraTS2016**	**90.11**
**YOLOv10 [proposed]**	**BraTS2016**	**84.37**
**YOLOv11[proposed]**	**BraTS2016**	**96.22**
**YOLOv9 [proposed]**	**Fig share Brain Tumor**	**90.12**
**YOLOv10 [proposed]**	**Fig share Brain Tumor**	**85.34**
**YOLOv11[proposed]**	**Fig share Brain Tumor**	**96.41**

Significant values are in bold.

Conventional methods achieve segmentation performance of 99.8% with the MIAS dataset, 99.34% with T1-weighted MRI, and 97.87% with BraTS datasets. Using the Figshare Brain Tumour Dataset, YOLOv11 achieved 96.41% accuracy, outperforming YOLOv9 (90.12%) and YOLOv10 (85.34%). YOLOv11 strikes the optimal balance between speed and precision, making it suitable for real-time brain tumour diagnosis and meeting clinical standards. A range of ML and DL methods for tumour classification and segmentation is explored, as shown in Fig. 18.

**Fig. 20:**
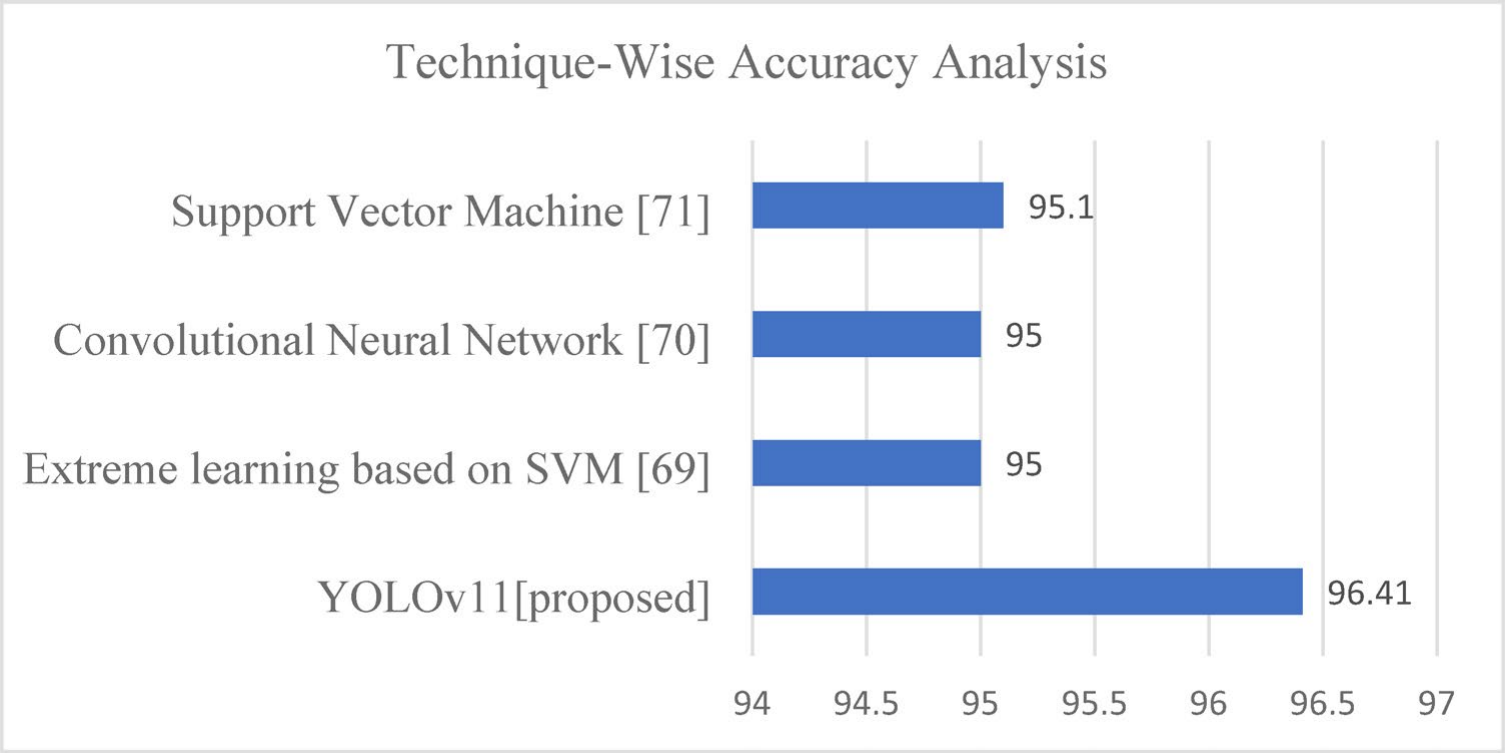
Comparative accuracy of traditional models and proposed YOLOv11.

DL models such as Support Vector Machines (95.1%), Convolutional Neural Networks (95%), and Extreme Learning based on SVM (95%) were examined. On the Figshare Brain Tumour Dataset, YOLOv11 outperformed YOLOv9 (90.12%) and YOLOv10 (85.34%). YOLOv11 excels in detection and real-time operation, surpassing traditional ML approaches are visualised in Fig. 20. YOLOv10 offers solid performance with a lightweight design but lower accuracy, making it suitable for low-resource applications. YOLOv11’s exceptional performance makes it the ideal choice for clinical brain tumour diagnosis systems, offering rapid, accurate diagnoses that improve patient outcomes.

A comparison of our YOLOv11 model with a number of current state-of-the-art designs is shown in Table 13. Even though models like Mask R-CNN and U-Net perform well in segmentation, they are not designed with real-time inference in mind. Higher contextual comprehension is possible using transformer-based techniques as Swin Transformer and Attention U-Net, but they come with a significant computational cost. YOLOv11, on the other hand, offers a special balance between speed and accuracy, retaining a real-time inference rate of 5.3 ms while attaining equivalent or superior performance on the same or similar datasets.

**Table 13:** Comparison with state-of-the-art brain tumor detection and segmentation models.

Model	Dataset	Metrics	Prediction time in ms	Inference
U-Net^85^	BraTS 2020	Accuracy: 89%	140	Good segmentation, low speed
Mask R-CNN^86^	Figshare	Accuracy: 94.2	160	Instance segmentation, 2-stage
Swin transformer^87^	BraTS 2021	Accuracy: 98.71	200	Context-aware, compute intensive
Regular U-Net, Upgraded U-net, and Attention U-Net^88^	BraTS 2021	Dice Score Coefficient of 0.3902, 0.6877, and 0,6534	180	High precision, slow
AlexNet^49^	Br35h	Accuracy: 98.79%	–	High accuracy, sensitivity
YOLOv11 (Proposed)	BraTS + Figshare	Accuracy: 99.2% mAP : 99.3%	5.3	Fast, real-time detection and segmentation

All models were compared based on performance reported on either the BraTS 2020 or Figshare datasets to ensure a consistent and fair benchmark.

A transfer learning-based framework for classifying brain tumours using EfficientNet-B4 was presented in a recent paper^48^. Their method demonstrated an impressive 99.87% accuracy rate and good performance metrics in terms of sensitivity, specificity, precision, and F1-score when evaluated on the Br35H dataset with considerable data augmentation.

Our suggested YOLOv11-based approach prioritises real-time detection and segmentation capabilities across integrated datasets (BraTS and Figshare), with a substantially reduced prediction time of 5.3 ms, even if their model outperforms in classification accuracy. Therefore, by addressing computational performance and multi-task integration of detection and segmentation, our work enhances such high-performing classification systems.

## Conclusion

### Summary

This study proposed a sequential DL framework for brain tumor identification and segmentation by integrating robust preprocessing techniques with advanced YOLO variants YOLOv9, YOLOv10, and YOLOv11. The preprocessing pipeline involved intensity normalization, histogram equalization, and edge-based ROI extraction to enhance the quality and diagnostic relevance of MRI slices. Among the tested models, YOLOv11 demonstrated superior performance, achieving a classification accuracy of 99.2%, an F1-score of 0.990, a mAP@0.5 of 0.993, and a minimal inference time of 5.3 ms, making it suitable for real-time clinical applications. These results underline the framework’s effectiveness in delivering precise tumor localization with high speed and accuracy.

### Limitations

Despite its promising performance, the framework presents certain limitations. First, the evaluation was conducted using only two publicly available datasets, which may not fully reflect the heterogeneity of real-world clinical data across diverse populations and imaging conditions. Second, the model currently operates only on 2D MRI slices and does not incorporate 3D volumetric data, limiting its spatial contextual understanding. Additionally, the approach has not been validated on other imaging modalities like CT or PET, which may be crucial in multi-disciplinary diagnostic settings. The preprocessing methods, although effective for brain MRI, may require significant adaptation for other anatomical regions or tumor types. Finally, clinical interpretability and explainability of the model decisions remain limited.

### Future work

Future research will focus on addressing these limitations by expanding the framework to support 3D volumetric MRI analysis and incorporating multi-modal MRI sequences, such as T1, T2, FLAIR, and contrast-enhanced images from datasets like BraTS. Model generalization and robustness will be assessed across larger and more diverse clinical datasets. Furthermore, we aim to optimize the YOLOv11 architecture for deployment on low-power edge devices, such as embedded GPUs and Raspberry Pi, enabling real-time diagnostics at the point of care. Efforts will also be directed toward integrating explainable AI techniques to enhance clinical interpretability, supporting better decision-making and fostering trust among medical professionals. Ultimately, our goal is to evolve the system into a comprehensive, real-time, and clinically interpretable tool for brain tumor analysis.

## Data Availability

The data that support the findings of this study are available from the corresponding author upon reasonable request.
